# Outreach, Screening, and Randomization of APOE ε4 Carriers into an Alzheimer’s Prevention Trial: A global Perspective from the API Generation Program

**DOI:** 10.14283/jpad.2023.27

**Published:** 2023

**Authors:** T. Walsh, L. Duff, M.-E. Riviere, P.N. Tariot, K. Doak, M. Smith, B. Borowsky, C. Lopez Lopez, P.C. Arratia, F. Liu, I. Scholten, D. Gordon, J. Arbuckle, A. Graf, M. Quinn, J. Ricart, J.B. Langbaum

**Affiliations:** 1. Banner Alzheimer’s Institute, Phoenix, AZ, USA; 2. Novartis Pharmaceuticals, East Hanover, NJ, USA; 3. Novartis Pharma AG, Basel, Switzerland; 4. Independent consultant to Novartis Pharma AG, Basel, Switzerland; 5. Novartis Farmaceutica SA, Barcelona, Spain

**Keywords:** Alzheimer’s disease, APOE, participant outreach, participant recruitment, participant screening, prevention trial, registries

## Abstract

**BACKGROUND:**

Alzheimer’s disease (AD) prevention trials require a large outreach and screening funnel to identify cognitively unimpaired adults who meet the study’s inclusion criteria, such as certain clinical or demographic criteria, genetic risk factors, and/or biomarker evidence of the disease.

**OBJECTIVES:**

Describe tactics and strategies to identify and enroll cognitively unimpaired adults with one (heterozygotes [HT]) or two (homozygotes [HM]) copies of the APOE ε4 allele, a genetic risk factor for dementia due to AD, into the Alzheimer’s Prevention Initiative (API) Generation Program, the largest and only prevention trials for late onset AD using this enrichment technique.

**DESIGN AND SETTING:**

The Generation Program was comprised of two global, randomized, double-blind, placebo-controlled, parallel group adaptive design with variable treatment duration clinical trials. Generation Study 1 randomized participants into one of two cohorts: Cohort 1 which evaluated CAD106 vs. placebo or Cohort 2 which evaluated umibecestat vs placebo. Generation Study 2 randomized participants into two doses of umibecestat vs. placebo. The Generation Program was terminated early in 2019, while enrollment was still occurring.

**PARTICIPANTS:**

Both Generation Study 1 and Generation Study 2 enrolled cognitively unimpaired APOE ε4 HMs aged 60-75; Generation Study 2 also enrolled APOE ε4 HTs ages 60-75 with elevated brain amyloid.

**METHODS AND MEASUREMENTS:**

Describe results of the centralized and localized outreach, recruitment, screening strategies and tactics as well as characteristics of sites successful at enrolling genetically eligible participants, with a particular focus on APOE ε4 HMs given the 2-3% prevalence of this genotype.

**RESULTS:**

At the time the trial program was terminated, 35,333 individuals had consented to the optional prescreening ICF1a/ICFA and provided a sample of DNA for APOE genotyping, 1,138 APOE ε4 HMs consented to screening for Generation Study 1 (ICF1b), and 1,626 APOE ε4 carriers were randomized into either Generation Study 1 or Generation Study 2. Genetic testing registries, partnerships with genetic testing/counseling companies, and the optional prescreening ICF1a/ICFA were the most successful strategies for identifying genetically eligible participants for screening.

**CONCLUSIONS:**

It is feasible to recruit, screen and randomize cognitively unimpaired APOE ε4 carriers, particularly APOE ε4 HMs for a global AD prevention trial. The Generation Program was on track to complete enrollment by end of 2019. Factors that were key to this success included: working with sites to develop customizable outreach, recruitment, and screening programs specific to their site needs, providing forums for sites to exchange best practices, and developing partnerships between the sponsor team and trial sites.

## Introduction

Alzheimer’s disease (AD) is the most common cause of dementia, accounting for 50-75% of all cases (1). Globally, there are an estimated 50 million people living with dementia, and close to 10 million new cases each year (2). There is a high unmet need for treatments that target the underlying disease pathophysiology at early stages, with the goal of delaying or preventing the onset of clinical symptoms due to AD. Longitudinal studies of initially cognitively unimpaired adults suggest that the underlying pathophysiology of the disease, beginning with amyloid beta (Aβ) plaque deposition followed later by neurofibrillary tau tangles, may occur before the onset of cognitive impairment (3, 4). This preclinical period, when there is biomarker evidence of disease without cognitive impairment, may be an optimal time to intervene with Aβ-lowering therapies (5, 6). Such prevention trials require a large outreach and screening effort to identify eligible participants (7–9).

The Alzheimer’s Prevention Initiative (API) Generation Program was a collaboration among the Banner Alzheimer’s Institute, Novartis, and Amgen, and supported partially by funding from several philanthropic organizations and a grant from the National Institute on Aging of the National Institutes of Health (NIH). The API Generation Program consisted of two trials, Generation Studies 1 and 2, both with the objective of delaying the onset of clinical symptoms of AD in participants at increased risk for dementia due to AD. Generation Study 1 enrolled cognitively unimpaired APOE ε4 homozygotes (HMs) ages 60-75; Generation Study 2 enrolled cognitively unimpaired APOE ε4 carriers (HMs and heterozygotes (HTs) ages 60-75, HTs were required to have elevated brain amyloid)(10). Participants underwent genetic testing, counseling and disclosure during screening as described previously (10, 11). Together the two trials aimed to enroll approximately 3,300 participants (approximately 1,340 in Generation Study 1, 2,000 in Generation Study 2). The Generation Program trials were terminated early, while enrollment was still occurring, when one of the treatments studied in the trials (the β-site amyloid precursor protein cleaving enzyme-1 [BACE-1] inhibitor umibecestat) was found to be associated with early mild cognitive worsening, as was observed with other BACE inhibitors (12, 13).

The API Generation Program required a large outreach, recruitment, and screening funnel to enroll cognitively unimpaired adults ages 60-75 who were APOE ε4 HM or HT (and if HT, also had elevated brain amyloid). Based on data from other observational studies, we expected that about 2-3% of those entering screening would be APOE ε4 HMs and 24-25% to be APOE ε4 HTs, of whom about half would have elevated brain amyloid (14–18). These numbers did not account for other inclusion or exclusion criteria, including but not limited to cognitive status, having a study partner, or contraindicated medications. As a result, the top of the screening funnel needed to be quite large, accommodating thousands of individuals and moving them efficiently through the screening process.

Here we describe the tactics and strategies and their success in the outreach, recruitment, screening, and enrollment of participants into the API Generation Program, with a specific focus on identifying and enrolling cognitively unimpaired APOE ε4 HMs given the 2-3% prevalence of this genotype in the population (16, 17). The API Generation Program was global, requiring a variety of outreach, recruitment, and screening approaches to accommodate differences across countries/regions as well as differing local laws and regulations regarding genetic testing, counseling, and disclosure. We also summarize key lessons learned that may be applicable to future AD prevention studies or trials for other indications that enroll based in part on the presence of one or more risk factors.

## Methods

### Study Design

**Figure 1.**
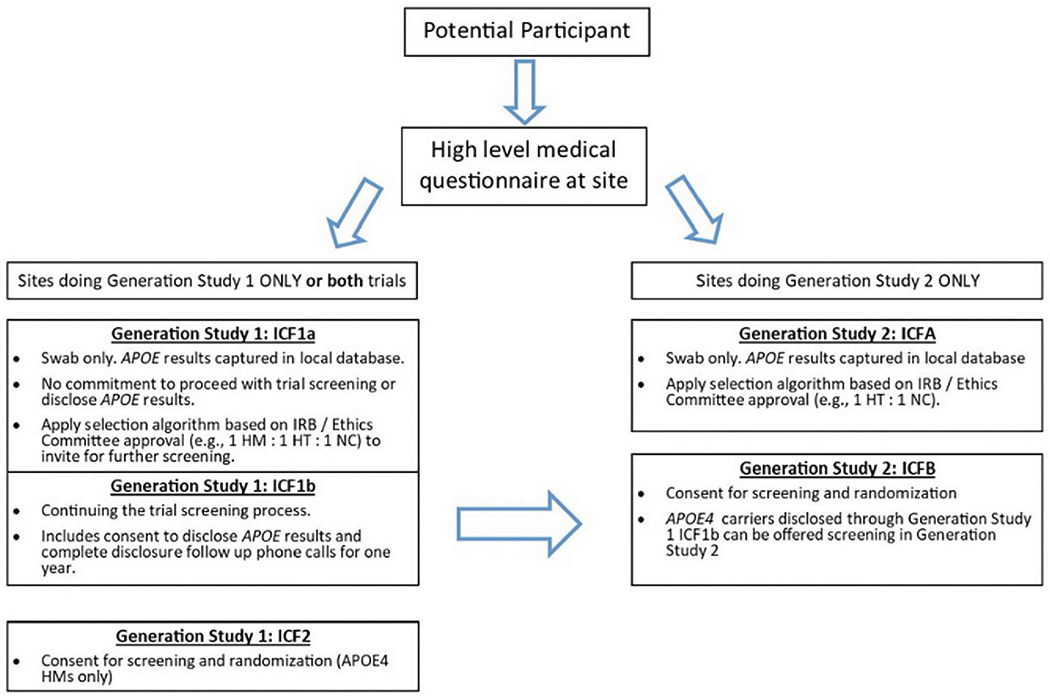
Participant Prescreening (ICF1a/ICFA) and Screening (ICF1b, ICF2, ICFB) Steps for Generation Study 1 and Generation Study 2

Generation Study 1 (ClinicalTrials.gov identifier NCT02565511) and Generation Study 2 (ClinicalTrials.gov identifier NCT03131453) were designed to assess the efficacy, safety, and biomarker effects of CAD106 and umibecestat in cognitively healthy adults at elevated risk for AD based on their age (60-75 years), APOE ε4 genotype and, for APOE ε4 HTs (APOE ε2/ε4 or ε3/ε4), the requirement of having elevated brain amyloid as measured by amyloid PET scan or cerebrospinal fluid (CSF) collected by lumbar puncture. The design and details of the screening processes, including genetic counseling and disclosure, have been described previously (10, 11). Figure 1 shows the prescreening and screening steps for each trial. The sponsor team encouraged sites to participate in both Generation Study 1 and Generation Study 2 to allow for synergies in outreach and recruitment since APOE ε4 HTs identified during screening for Generation Study 1 could be referred for screening in Generation Study 2. Results from both trials, including characteristics of the participants, were presented at the Alzheimer’s Association International Conference (19, 20). The trials had Institutional Review Board (IRB) / Ethic Committee (EC) approval and written informed consent for participation in the trial and for use of the data was provided by participants prior to any research activities being performed.

### Centralized Participant Recruitment Efforts / Sponsor-led Recruitment Support

**Figure 2.**
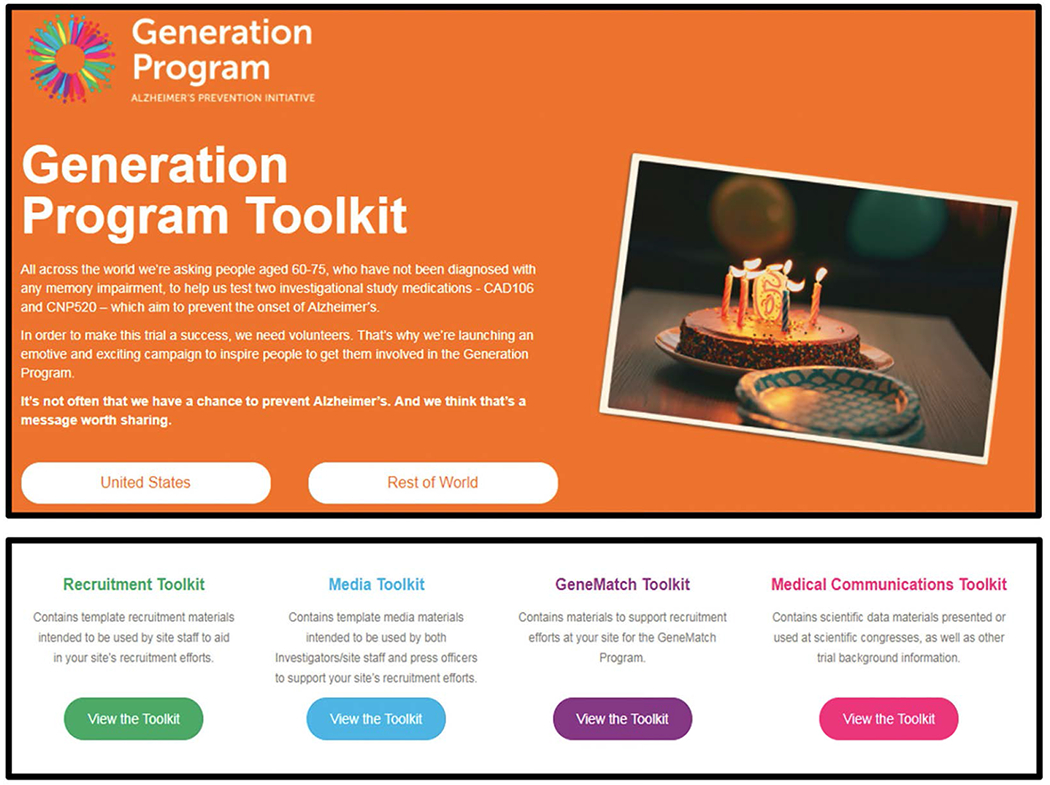
Generation Program Online Toolkit for Study Sites (United States version)

The Generation Program was a collaborative effort between Novartis, Amgen, and Banner Alzheimer’s Institute. A dedicated team including multiple representatives from each organization was formed and charged with managing the outreach and recruitment efforts. This team included clinical scientists, clinical trialists with considerable expertise being site investigators, researchers with expertise in registries and participant recruitment, project managers, clinical operations specialists, and site relationship and protocol medical/execution support managers. Table 1 provides examples of outreach and recruitment activities used in the Generation Program. A centralized recruitment company was retained in 2017 to develop a study website (available in multiple countries with local adaptations as necessary), assist with study advertising (print, internet/web, and social media), assist sites with local outreach events (e.g., booths at community walks), and coordinate travel concierge services for participants who resided far from their study site. The sponsor team also retained two PR agencies (one limited to United States [US], the other on all other countries enrolling in the Generation Program) with specialization in healthcare to assist with securing media coverage nationally and in selected local markets. The PR agencies were intended to complement and assist with media-related outreach and awareness activities conducted by the sites. The PR firm focused on US activities developed a study specific, password protected online toolkit that contained all study level recruitment materials developed for the Generation Program, making materials available for site personnel to download and customize with their site-specific information or translation in their local language (Figure 2). The sponsor team also partnered with patient advocacy groups, direct-to-consumer genetic testing and/or counseling companies, and participant recruitment registries to share information about the Generation Program with potentially eligible participants. In late 2018, the sponsor team formed a partnership with an advocacy group in the US to develop customized plans for outreach and engagement to traditionally underrepresented communities, with particular focus on Black/African Americans and Hispanic/Latinos.

Since there was not a “one size fits all” recruitment plan for deploying these materials that would work for all countries or study sites, representative form the sponsor team focused on building relationships with individual sites to understand their site-specific needs. This involved one-on-one calls and, in some cases, in-person visits to the sites soon after site activation to help develop and implement customized recruitment plans specific to each site’s needs and capabilities. In many cases, these individualized interactions continued throughout the recruitment period, allowing the sponsor team and site personnel to further refine outreach and recruitment activities and provide additional targeted support to sites to meet their recruitment and enrollment goals. Examples of activities and tactics included development or implementation of site-specific recruitment ads, genetic testing “swabbing” events at which consent was obtained to collect genetic samples from a large number of individuals at one time (21), staffing booths at local Alzheimer’s walks, and creating recruitment videos. Sites were empowered to direct interested individuals to either a local or national registry or use the optional ICF1a/ICFA prescreening consent (described in greater detail below) depending on the site’s preferences and staffing capabilities.

The sponsor team provided opportunities for site personnel to share their outreach and screening experiences identifying and enrolling APOE ε4 carriers (often with an emphasis on APOE ε4 HMs, given the low [2-3%] prevalence of the genotype), spurring new ideas and forming collaborations among sites. In the US, the sponsor team held six regional roundtable dinner meetings during which representatives from 10-12 nearby sites discussed recruitment activities. Attendees included site principal investigators, study coordinators, recruitment / outreach specialists as well as representatives from the sponsor team. The sponsor team also held two larger, one-day recruitment and retention focused workshops in the US and three in Europe. Representatives from all sites in these regions were invited to these workshops along with members of the sponsor team. Principal investigators (PIs) from sites with high screening and enrollment numbers were invited to speak at the roundtables and workshops to share their strategies and tactics with other sites. The regional roundtable meetings and one-day workshops also provided a venue to discuss protocol and scientific questions with the sponsor team.

The sponsor team held regular country-specific teleconferences with site personnel from that region to discuss outreach, recruitment, screening, and retention, answer frequently asked questions about participant prescreening and the protocol, clarify study memos, and any other questions from site teams in real time. These were complemented by monthly “media and communications support” teleconferences led by the PR firm or sponsor team that provided tips for sites to engage with their local media and strategies for using social media for recruitment. All sites received weekly newsletters with study updates, enrollment metrics, and other pertinent, time-sensitive information.

### Participant Prescreening and Screening

An optional ICF1a/ICFA prescreening program was created to aid sites that did not have access to a local or national registry with APOE genotyped individuals. Additionally, this program was beneficial for sites that preferred to handle the prescreening process themselves as it provided them more direct control compared to the multi-step course of referring interested individuals to outside local or national registries for genetic testing and, if genetically eligible, wait to receive an invitation to the Generation Program. This prescreening program provided sites with the flexibility to create their own local registries from which they could invite individuals for further screening into the Generation Program or other trials at their site. The prescreening process was described in brief informed consents (ICF1a for Generation Study 1, ICFA for Generation Study 2) (Figure 1). In some countries, sites were also able to receive referrals from local or national registries that collected APOE genotype information and refer interested individuals for screening into the Generation Program, such as GeneMatch in the US (22).

Sites stored the demographic information from participants who consented to ICF1a/ICFA and did not share the information with the sponsor. APOE genotype results obtained from the ICF1a/ICFA process were returned directly to the study site. Sites used the APOE results to determine which participants to invite for further screening (ICF1b or ICFB shown in Figure 1) based on approval from their IRB / EC, ensuring that the invitation itself did not inadvertently disclose APOE results (i.e., inviting a ratio of genetically eligible to non-eligible for screening via IFC1b/ICFB). APOE genotype obtained from the ICF1a/ICFA testing was only shared with the sponsor and included in the clinical database if the participant was eligible for and consented to further screening for the trial (ICF1b/ICFB). The optional prescreening ICF1a/ICFA consents were implemented in all countries except Italy, Japan, and Portugal due to requirements to disclose genetic results to all participants who provided a DNA sample. When an IRB / EC or site determined it was necessary to offer all participants genetic disclosure, counseling and disclosure were performed outside the context of the study and all safety related follow up was the responsibility of the PI. Sites participating in both Generation trials were advised but not required to consent participants for Generation Study 1 using ICF1b and from there, route all disclosed APOE ε4 HTs to Generation Study 2 for further screening using ICFB. Results will focus primarily on Generation Study 1 given the 2-3% prevalence of the APOE ε4 HM genotype.

## Results

**Figure 3.**
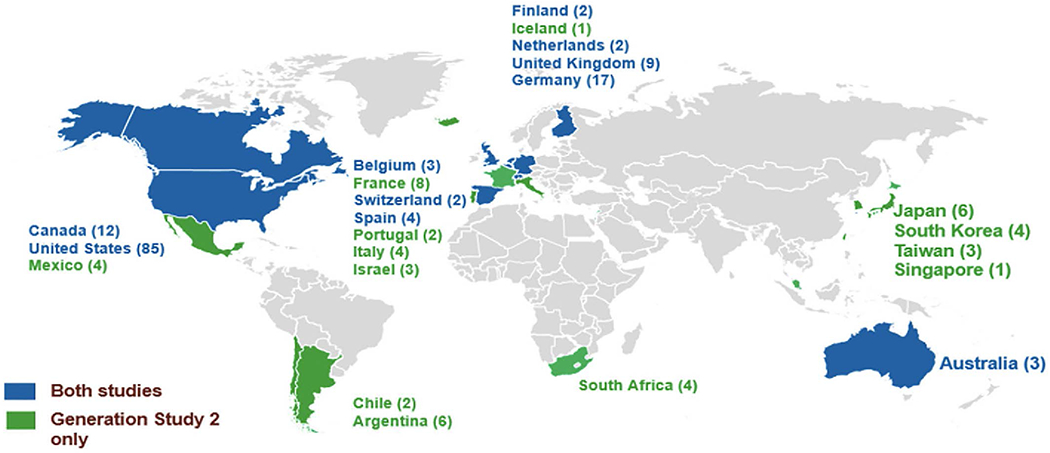
Locations of Generation Program Trial Sites Number of study sites in a country shown in parentheses next to country name

Recruitment and enrollment into the API Generation Program was terminated in July 2019 after an early signal of mild worsening in some measures of cognitive function with umibecestat (19, 20). At the time the trial program was terminated, there were 207 sites actively recruiting in 23 countries (13 countries participated in Generation Study 2 only) and over half of the sites were participating in both trials (Figure 3). At the time of termination, 35,333 participants had consented to ICF1a/ICFA prescreening for APOE genotyping (11,883 in the US) across both trials, 9,623 screened (including referral from other sources [e.g., registries]) and 1,626 were randomized into either Generation Study 1 or Generation Study 2. The program was expected to complete enrollment by end of 2019.

The online toolkit was launched in January 2018 and deactivated in July 2019 following the early termination of the Generation program. The most frequently downloaded materials for use after EC/ IRB approval were (1) recruitment brochure (266 downloads), (2) recruitment ads (238 downloads), (3) recruitment posters (230 downloads), (4) recruitment ‘share sheet’ (207 downloads), and (5) trial talking points (181 downloads).

Table 1 includes sponsor insight to select outreach and recruitment activities used in the trial program. The Generation Program website went live in the US in October 2018 and was deactivated in July 2019 when the trials were terminated. Prior to website launch, the centralized recruitment vendor relied primarily on email outreach and paid online advertising to raise awareness about the program. People were most likely to use a mobile device to visit the trial website (44.8%), followed by using a tablet device (35.9%). Social media advertising drove most of the traffic to the website (47%). There were 141,119 total website sessions that resulted in 1,981 pre-screen attempts and 380 referrals to study sites. The centralized recruitment vendor assisted 34 sites with 37 events and community walks between September 2018 and January 2019. Together these events were attended by over 48,500 people. Genetic testing (swabbing) was done at seven events, swabbing a total of 289 people, and resulting in three randomizations. Data on the number of APOE ε4 carriers identified at these events are not available. The centralized recruitment vendor was responsible for 20 randomizations in the US; 21 referrals (11 in the US, 10 in Australia) were still in screening at study sites as of July 2019. Between October 2017 and April 2019, the recruitment vendor referred 17,080 people to GeneMatch. Of these, by June 2019, 4,009 (26%) consented to GeneMatch of whom 2,444 (61%) returned their completed swabs to the GeneMatch lab for processing. Of these, 556 had been invited by GeneMatch to screen for the Generation Program, 210 accepted their invitation, and 87 had enrolled/consented to screening. The centralized recruitment vendor assisted 10 sites in the US with a mailed letter campaign to nearby healthcare providers (HCPs). These letters contained general information about the Generation Program and contact information for a nearby site. The HCP could refer their patient(s) to the nearby site for screening. A total of 667 letters to HCPs were sent between November 2018 and February 2019. Six HCPs responded that they would be interested in referring their patients to the trial, five HCPs declined, four HCPs responded ‘maybe’; 38 letters were returned as undeliverable. This outreach did not result in any randomizations.

Roundtable meetings were held in 6 US cities (Chicago, Ft. Lauderdale, Houston, Los Angeles, Philadelphia, and Phoenix); 80 trial site personnel representing 35 sites participated in these. Two recruitment/retention focused meetings were held in the US (Atlanta and San Francisco) in April 2019 attended by 77 trial site personnel. One recruitment/retention focused meeting was held in Canada (Toronto) in May 2019 attended by 22 site personnel. Three recruitment/retention focused were held in Europe (Amsterdam, London, and Madrid) March-May 2019 attended by 90 trial site personnel representing 56 sites. In addition to large meetings, members of the sponsor team visited individual study sites and held calls with site personnel to discuss site-specific recruitment and retention needs.

The US-focused PR agency helped secure media coverage including feature articles in several national publications, e.g., The Wall Street Journal, USA Today, and the Associated Press (which also included a video). The USA Today article resulted in 7.9 million impressions. The Associated Press article was syndicated in 42 additional outlets and resulted in 24.7 million impressions. The Associated Press article contributed to 414% increase in signups to the Alzheimer’s Prevention Registry and its GeneMatch program in the weeks following the article publication compared to the weeks prior without media coverage. In local US markets, the PR agency helped place 109 articles/stories during 2018, garnering 6.8 million impressions, leading to increased awareness and referral inquiries to study sites. For example, a television interview that aired on a local affiliate station resulted in 80 telephone calls to the nearby study site following the interview. Data are not available to determine if these media efforts resulted in people screening for the trial or randomizations, nor are data available for media coverage outside of the US.

At the time the trial program was terminated, 35,333 individuals had consented to ICF1a/ICFA and provided a sample of DNA for APOE genotyping. Tables 2a and 2b display participant demographics and the prescreening source of the initial APOE genotyping which was then used to invite individuals for further screening for Generation Study 1 (IC1b), in which only APOE ε4 HMs were eligible for randomization. Tables 2a and 2b separate data from the US from all other countries (which are grouped together), since GeneMatch and direct-to-consumer genetic testing/genetic counseling referrals were only available in the US. In the US, GeneMatch was the single largest prescreening referral source of APOE ε4 HMs who consented for further ICF1b screening (37.7%), followed by direct-to-consumer genetic testing/genetic counseling companies (35%) (Table 2a). In the US, the ICF1a/ICFA prescreening identified 16% of the APOE ε4 HMs who consented for ICF1b screening. Outside of the US, the optional ICF1a/ICFA prescreening process identified nearly all the APOE ε4 HMs (87.2%) who consented for further ICF1b screening, with 7.6% coming from national or local registries (Table 2b). Table 3 displays the breakdown of participants invited for further screening for Generation Study 1 (IC1b) by country. It is important to note that countries began enrolling at different times.

## Discussion

Many AD prevention trials require participants to have a genetic risk factor and/or biomarker evidence of disease. A risk factor with low prevalence, such as APOE ε4 homozygosity which is present in only 2-3% of the general population (approximately 4-5% of those with a first-degree family history of dementia) (18), will likely in a high screen fail rate. The challenge of identifying such a small proportion of a suitable population who do not have a diagnosis of cognitive impairment and who are likely not yet connected with a memory specialist presented an opportunity to deploy a broad range of strategies and tactics to assist with recruitment. The current results suggest that it is possible to recruit, screen, and enroll cognitively unimpaired APOE ε4 HMs within a reasonable timeline for a global AD prevention trial. This is noteworthy since APOE genetic testing and disclosure is not currently part of routine medical care.

The API Generation Program used a variety of recruitment and screening approaches, recognizing that there was not a “one size fits all” plan that could be applied to all countries and sites. This flexible, tailored approach was also found to be successful for a high-volume, non-interventional observational cohort study (23). Developing partnerships between the sponsor team and each site while allowing sites to select from a menu of outreach and screening strategies and tactics to customize their plans in a manner that capitalized on their strengths and the needs of their catchment area was a process that was received well by site personnel. The strategies and tactics that were most likely to result in raising awareness and interest in screening for the Generation Program included: earned media coverage, especially when national stories could be leveraged locally, social media advertising, memory screening events that engaged the “worried well” and allowed the sites to develop or maintain a relationship with those screened in a manner approved by their IRB/EC, and mass mailings of postcards/brochures that contained a call to action to call (or in some cases, email) the site directly. The mailings were most successful if the site was able to speak promptly with the person or within 1-2 days of call/email. The strategies that were most likely to identify APOE ε4 carriers, especially APOE ε4 HMs, for screening included: partnerships with genetic testing registries (such as GeneMatch) or other local registries, direct-to-consumer genetic testing / counseling companies, and implementation of the optional ICF1a/ICFA prescreening process. It is difficult to track the success of the centralized recruitment efforts undertaken by the recruitment vendor to identify genetically eligible participants for the trial program. Anecdotal feedback from the sponsor team identified characteristics of sites that were successful in randomizing large numbers of participants including: having dedicated recruitment specialists/outreach coordinators who knew what activities worked well in their communities, being proactive in community outreach, such as conducting community events targeting healthy adults on a regular basis, and targeting their prescreening activities, including proactively exerting efforts to understand if the individual was a good fit for a clinical trial, having adequate staffing for pre-screening activities at the site (phone or in-person activities), and using prescreening checklists.

The API Generation Program developed the ICF1a/ICFA prescreening program to support sites that did not have access to a local or national registry with APOE genotyped individuals or that preferred to handle the prescreening process themselves rather than refer interested individuals to registries. Although there are registries outside of the US that contain APOE results, few are set up like GeneMatch, which has the specific objective to connect with and invite its members to AD-focused studies based in part on their APOE results. In the US, sites differed in their perceptions of the ICF1a/ICFA prescreening option. Some indicated that the ICF1a/ICFA was more efficient and allowed them to develop their own registry at relatively low cost (to the site), whereas other sites preferred to direct interested individuals to programs such as GeneMatch and dedicate their staffing to the trial itself rather than the prescreening program. Some sites perceived GeneMatch to be inefficient and/or that it operated in manner that was not fully transparent to them. Globally, even sites were successful at consenting large numbers to the ICF1a/ICFA prescreening process and identifying APOE ε4 HMs experienced challenges at moving these participants to the next steps of screening (ICF1b/ICFB) and randomization. Sites did not share with the sponsor team the reason(s) for the lack of interest from these genetically eligible individuals (not all of whom were aware of their APOE genotype): we postulate that it may have been due the lag time between screening steps, lack of interest in joining a clinical trial of this nature, and/or some individuals may have used the prescreening process chiefly as a means of learning the APOE genotype.

We acknowledge several limitations. The discussion and conclusions provided here are based on the data available at the time of termination. Had the program continued to the end of 2019 (when recruitment was projected to be complete), other strategies or tactics may have become more successful, and countries may have identified more APOE ε4 HMs. For instance, perhaps the ICF1a/ICFA prescreening in the US may have referred more APOE ε4 HMs for further screening, as sites implemented new outreach and recruitment strategies that were shared at the roundtables and recruitment meetings. Similarly, the partnership with advocacy groups to promote enrollment of individuals from traditionally underrepresented groups was implemented in spring 2019, just a few months before the trial was terminated. As a result, there are no metrics to describe the success of this effort. Another limitation is the scarce data available to retrospectively analyze the details of the ICF1a/ICFA prescreening program since data were entered into site databases rather than a central, study-level database. Without this data it is impossible to compare the characteristics of individuals who participated in the ICF1a/ICFA prescreening program to those in local or national registries, such as GeneMatch. Furthermore, sites were not consistent in tracking and reporting metrics associated with each recruitment strategy and tactic deployed at their site. In the future, sponsors may want to develop tracking systems that allow sites to easily collect and report referral sources, reasons for prescreening ineligibility, and other important metrics. Although we know the source of initial APOE genotyping (e.g., ICF1a/ICFA, GeneMatch, direct-to-consumer, etc.) at a study level for participants who consented to the disclosure step, we lack such information for participants who were not invited (i.e., noncarriers) or APOE ε4 carriers who did not respond to invitation for disclosure. It is not possible to know which tactic (e.g., newspaper article, trial website, word of mouth) alerted the participant to the trial and motivated their consent for APOE genotyping. Metrics were not collected systematically for many strategies and tactics, limiting our ability to draw specific conclusions about what works best at the site level (e.g., small-group community informational sessions compared to one-on-one sessions (24)). It is not possible to calculate and compare the cost-effectiveness of the different strategies and tactics used due to differences in tracking across organizations. Future efforts should consider using a central database to track this information. Lastly, because the trial program was terminated early, it remains unknown whether route of initial enrollment (e.g., ICF1a/ICFA prescreening, registry, genetic testing/counseling company) was associated with better participant retention in the trial.

Despite these and other limitations, we demonstrated that it is feasible to develop outreach and screening programs for cognitively unimpaired adults to identify and enroll APOE ε4 carriers, particularly APOE ε4 HMs, into a global AD prevention trial. The Generation Program was on track to complete enrollment by end of 2019. Genetic testing registries (such as GeneMatch in the US), partnerships with genetic testing/genetic counseling companies, and the optional prescreening ICF1a/ICFA were the most successful strategies for identifying genetically eligible participants for screening, though the success of the ICF1a/ICFA prescreening process was greater outside of the US. The sponsor team recognized that a universal “one size fits all” recruitment plan would not be effective due to local, regional, and country-level differences. Collaborations between the sponsor team and individual trial sites were effective in supporting sites to be successful with their participant outreach, recruitment, and randomization goals. Frequent phone and email communication between the sponsor team and sites that was augmented by in-person meetings were instrumental in developing and maintaining the collaborative partnerships. The Generation Program provides a roadmap for developing and implementing outreach, recruitment, screening, and enrollment processes that may be applicable to future AD prevention studies or trials for other indications enrolling healthy adults based in part on the presence of one or more risk factors.

## Figures and Tables

**Table 1.** Examples of Outreach and Recruitment Activities Used in the Generation Program

Activity	Description	Examples	Sponsor Team Insights
Community outreach/events	Site initiated outreach to their community; direct contact with community members intended to inform people about prevention research/genetic testing/Generation Program. Some sites received assistance from the centralized recruitment vendor.	Principal investigator (PI) speaking event; memory screening, ICF1a/ICFA screening events, GeneMatch swab events, walks, health fairs	Very helpful in raising awareness and engaging the community, especially if tied to an immediate action (e.g., ICF1a/ICFA, GeneMatch swab) to join a local or national registry/database
Paid media	Site initiated paid advertisements using either sponsor created materials or site materials approved by sponsor and IRB/EC.	Newspaper, radio and TV ads, billboard signs, bus stop ads	Used primarily in the US. May have helped raise awareness locally but difficult to track success
Earned media	Unpaid national, regional and local media coverage that promoted a particular site (or sites’) involvement in the Generation Program.	Editorial, radio/TV interview of PI or other site staff with local media	Important for media coverage to include contact information for site. Very helpful in raising awareness, most successful if site responds to inquiries within 24hrs.
Social media	Paid and boosted ads on social media sites, led by study sites. All materials approved by sponsor and IRB/EC.	Facebook advertisements and Facebook page (site specific)	Limited success (it was very successful when used nationally to promote registries). One caveat to note, the proprietary algorithm used to determine who sees the ads may reduce ethnic, racial, and sex/gender diversity.
Registry or database	Pre-existing registries/local databases designed to connect enrollees with clinical studies and informed consent allows for re-contact for potential clinical trial participation. These may or may not have previously disclosed APOE genotype information.	GeneMatch, DecoDE, BarcelonaBeta, Chariot Registry, AURIA Biobank	Extremely helpful and efficient at identifying genetically eligible individuals. Confusion arises when interested participants are told they need to join the Registry and then may be contacted to screen for the trial. Information about the clinical trial opportunity needs to be communicated earlier and more explicitly.
Centralized recruitment activity	Centralized recruitment activities through a sponsor-retained recruitment vendor. All materials available to all sites to request, download, print as needed. Sites initiated activities and tactics most relevant/helpful to them.	Print materials (pre-screening checklist, inclusion/exclusion checklist, Lay summary, recruitment letter, brochure, posters, ads, sharesheet), digital marketing campaigns, email outreach campaign, study website, social media, call center, concierge travel assistance, HCP letters	Postcard mailings extremely successful when they included name/logo of recognizable institutions (e.g., major university or hospital). Some tactics were difficult to track success (may have helped raise awareness generally). Access to the tools alone did not lead to their use; experienced sites knew what worked in their community, other sites needed encouragement to use tools.

**Table 2a.** Demographics and Source of Initial APOE Results from Participants Screening for Generation Study 1 (ICF1b) – United States ONLY

	APOE ε4 Homozygotes (n = 740)	APOE ε4 Heterozygotes (n = 2298)	APOE ε4 Non-carriers (n = 1017)	Total (n=4055)
Age, mean (SD)	66.0 (4.2)	68.21 (3.9)	68.1 (4.1)	67.8 (4.1)
Female (%)	62.8	63.7	63.8	63.6
Education years, mean (SD)	16.6 (2.7)	16.4 (2.9)	16.4 (2.8)	16.5 (2.8)
Family history of AD (%)				
parent	67.4	68.9	58.4	66.0
sibling	3.0	3.2	2.9	3.1
grandparent(s)	6.1	5.6	8.4	6.4
other	5.5	4.1	6.7	5.0
None	18.0	18.2	23.7	19.6
Race (%)				
White	92.8	92.3	93.8	92.8
Black	4.2	3.6	2.5	3.4
Asian	0.7	0.4	0.9	0.6
Native American	0	<0.1	0	<0.1
Pacific Islander	0.1	0.1	0	<0.1
Other	0.1	0.3	0.5	0.3
Unknown	2.0	3.2	2.5	2.8
Ethnicity (%)				
Hispanic or Latino	0.7	0	<0.1	<0.1
South Asian	0	<0.1	0	<0.1
Southeast Asian	0	<0.1	0	<0.1
West Asian	0.1	0	0.1	<0.1
Other East Asian	0	0	0.2	<0.1
Russian	0.5	0.5	0.6	0.5
Japanese	0.3	0.2	<0.1	0.2
Chinese	0.3	0.1	0.2	0.2
Mixed ethnicity	11.2	10.1	8.5	9.9
Other	53.2	49.7	54.0	51.4
Unknown / not reported	33.7	37.3	33.2	35.6
Source of genotype n (%)				
Direct-to-consumer genetic testing	259 (35%)	32 (1.4%)	2 (0.2%)	293 (7.2%)
ICF1a / ICFA	119 (16%)	857 (37.3%)	358 (35.2%)	1334 (32.9%)
GeneMatch	279 (37.7%)	1148 (49.9%)	569 (55.9%)	1996 (49.2%)
Local Registry	26 (3.5%)	156 (6.8%)	47 (4.6%)	229 (5.6%)
Other / unknown	57 (7.7%)	105 (4.6%)	41 (4.1%)	203 (5%)

**Table 2b.** Demographics and Source of Initial APOE Results from Participants Screening for Generation Study 1 (ICF1b) – all countries EXCEPT United States

	APOE ε4 Homozygotes (n = 398)	APOE ε4 Heterozygotes (n = 2753)	APOE ε4 Non-carriers (n = 2858)	Total (n=6009)
Age, mean (SD)	66.89 (4.1)	67.3 (4.0)	67.2 (4.0)	67.2 (4.0)
Female (%)	52.5	59.8	59.6	59.2
Education years, mean (SD)	15.3 (4.0)	15.3 (4.0)	15.3 (3.9)	15.3 (4.0)
Family history of AD (%)				
parent	63.1	56.7	40.0	49.2
sibling	4.0	3.3	1.8	2.6
grandparent(s)	3.3	5.0	5.1	5.0
other	4.8	5.0	5.2	5.1
None	24.9	30.0	47.9	38.2
Race (%)				
White	92.7	91.6	96.0	93.7
Black	0.3	0.3	0.2	0.2
Asian	0.5	0.6	1.0	0.8
Native American	0	0	0	0
Pacific Islander	0	0	0	0
Other	1.0	0.7	0.7	0.7
Unknown	5.5	6.9	2.1	4.5
Ethnicity (%)				
Hispanic or Latino	0.3	0.2	<0.1	0.1
South Asian	0	0.1	0.5	0.3
Southeast Asian	0	<0.1	0.2	0.1
West Asian	0	0	0	0
Other East Asian	0	<0.1	0	<0.1
Russian	0	0	0	0
Japanese	0	0	<0.1	<0.1
Chinese	0.3	0.1	<0.1	<0.1
Mixed ethnicity	1.8	2.1	0.4	1.3
Other	82.7	77.0	87.1	82.2
Unknown / not reported	15.1	20.3	11.7	5.9
Source of genotype n (%)				
Direct-to-consumer genetic testing	0 (0%)	4 (0.2%)	1 (<0.1%)	5 (<0.1%)
ICF1a / ICFA	299 (87.2%)	2348 (94.1%)	2215 (81.4%)	4862 (87.5%)
GeneMatch*	1 (0.3%)	2 (<0.1%)	0 (0%)	3 (<0.1%)
Local Registry	26 (7.6%)	83 (3.3%)	16 (0.6%)	125 (2.2%)
Other / unknown	17 (4.9%)	57 (2.3%)	490 (18%)	564 (10.1%)

* GeneMatch only available in the United States. Site(s) likely selected this option in error.

**Table 3.** Participants Screening for Generation Study 1 (ICF1b) by Country

	APOE ε4 Homozygotes	APOE ε4 Heterozygotes	APOE ε4 Non-carriers	Total
Australia	10	96	13	119
Belgium	3	22	12	37
Canada	55	259	136	450
Finland	27	139	4	170
Germany	28	99	26	153
Great Britain	205	1697	2077	3979
Netherlands	26	49	35	110
Spain	34	293	529	856
Switzerland	10	99	26	135
United States	740	2298	1017	4055
